# Risky Sexual Behavior, Paraphilic Interest, and Sexual Offending: The Study of a Community Sample of Young Adults in Hong Kong

**DOI:** 10.3390/ijerph20054279

**Published:** 2023-02-28

**Authors:** Heng Choon (Oliver) Chan, Wade C. Myers

**Affiliations:** 1 Department of Social Policy, Sociology, and Criminology, School of Social Policy, University of Birmingham, Birmingham B15 2TT, UK; 2 Division of Forensic Psychiatry, Warren Alpert Medical School of Brown University, Providence, RI 02903, USA

**Keywords:** sexual offending, sexual offense, perpetration, risky sexual behavior, paraphilic interest, Hong Kong

## Abstract

Limited information is available on the prevalence and nature of sexual offending in Hong Kong. This cross-sectional study seeks to explore the role of risky sexual behavior (RSB) and paraphilic interests in self-reported sexual offending behavior (i.e., nonpenetrative-only, penetrative-only, and nonpenetrative-plus-penetrative sexual assault) in a community sample of young adults in Hong Kong. Using a large sample (*N* = 1885) of university students, the lifetime prevalence of self-reported sexual offending was 18% (*n* = 342; 23% males (*n* = 166), 15% females (*n* = 176)). Based on the study subsample of 342 participants who self-reported sexual offending (aged 18–35), the findings indicated that males reported significantly higher levels of general, penetrative-only, nonpenetrative-plus-penetrative sexual assault; and paraphilic interest in voyeurism, frotteurism, biastophilia, scatophilia, and hebephilia than females; while females reported a significantly higher level of transvestic fetishism than males. No significant difference was found in RSB between males and females. Logistic regressions found that the participants who possessed a higher level of RSB, particularly penetrative behaviors, and paraphilic interest in voyeurism and zoophilia were less likely to engage in a nonpenetrative-only sexual offense. Conversely, the participants who possessed higher levels of RSB, especially penetrative behaviors, and paraphilic interest in exhibitionism and zoophilia, were more likely to engage in nonpenetrative-plus-penetrative sexual assault. The implications for practice in areas such as public education and offender rehabilitation are discussed.

## 1. Introduction

Sexual victimization occurs in all ages, sexes, ethnicities, educational fields, and socioeconomic groups. It is clear that sexual offenses are serious incidents and widely recognized as both a violation of human rights and a public health concern worldwide. Sexual offending is broadly defined as any nonconsensual sexual act perpetrated against another person that may cause unwanted physical or psychological harm [1]. Sexual offending covers a range of criminal sexual acts, including sexual assaults of older adolescents or adults, sexual contact with children under the legally defined age of each jurisdiction, noncontact sexual offenses involving exhibitionism or voyeurism (e.g., this could include online offending behaviors or use of video/camera devices to capture images of unsuspecting victims), and engagement with illegal pornography (e.g., child pornography, revenge pornography) [2]. Sexual acts include overt physical contact (or attempted contact) that targets erogenous zones or actions that are generally sexually motivated, and verbal statements that indicate intentions to commit these acts [3]. Victims (The term “victims” used in this study refers to both victims and survivors.) often experience a wide array of physical, psychological, social, occupational, and financial costs following their sexual victimization [1].

Sexual offending, in general, is committed under different circumstances and contexts, such as illegal sexual penetration (i.e., rape) and unwanted sexual contact (e.g., sexual molestation). Its incident rates often vary from country to country as a result of different sociocultural factors, reporting practices, and laws [1]. The nature of sexual offending can be instrumental (i.e., it occurs when an offender is interested in obtaining something that he or she currently lacks but which is possessed by another person) or expressive (e.g., an emotional reaction commonly brought about by anger or provocation, and the ultimate aim is to retaliate and inflict pain on the victim) [4,5]. Additionally, past research has demonstrated that the offenders’ demographic characteristics, including sex [6], age [7], religiosity [8], marital status [9], and education [10], are significantly correlated with their propensity, type, severity, and dynamics in perpetrating sexual offending behavior.

Research on sexual offending has largely been conducted with official records (e.g., police data), clinical data (e.g., clinical interviews), and community surveys (e.g., self-reported data). The World Health Organization’s (WHO) Multi-Country Study on Women’s Health and Domestic Violence Against Women analyzed data from multiple sites in 10 countries (Bangladesh, Brazil, Ethiopia, Japan, Namibia, Peru, Samos, Serbia, Tanzania, and Thailand) and found that the lifetime prevalence of sexual intimate partner violence (IPV; e.g., marital rape) ranged from 6% to 59% [11]. In addition to sexual IPV, the WHO study [12] based on cross-national data from more than 80 countries found that 7% of women worldwide reported having been sexually assaulted by someone other than an intimate partner. As indicated in both official records and community surveys, the majority of sex offenders are men, with many of them having committed their first sexual offense as a juvenile [13]. Cortoni and Hanson [14] suggested that the ratio of male to female sex offenders was about 20:1 and thus female sex offenders were only responsible for approximately 5% of all sexual assaults. Of note, research on sexual offending in East Asia has mostly involved intimate partners as the offenders (i.e., sexual IPV), while only a handful of studies have examined sexual offending committed by acquaintances or strangers. In East Asia, South Korea had the highest prevalence of sexual violence in 2016, with 44 cases per 100,000 women compared with six cases in Japan, 14 cases in Macau, and 10 cases in Mongolia [15]. Specifically in Hong Kong, the arrest rates for rape fluctuated over the past decade, with 121 police arrests made in 2012, 65 arrests in 2017, and 79 arrests in 2021 [16]. However, a downward trend was observed in police arrests made on indecent assaults (e.g., sexual molestation), with 1495 arrests made in 2012, 1077 arrests in 2017, and 1018 arrests in 2021. It should be noted that sexual offenses are underreported; hence, the actual sexual offense statistics are not reflected in the reporting rates.

Studies on sexual offending risk factors have been primarily focused on offenders from Western countries [17,18,19]. Limited information is available about the prevalence and risk factors of sexual offence perpetration in Hong Kong; consequently, this area is a gap in the literature and warrants further investigation. Of note, sexually deviant behavior and/or risky sexual behavior (RSB) are perceived as mental health conditions or psychiatric disorders in this study. This study adds cultural and geographical diversity to the literature by drawing from an under-researched population, i.e., Hong Kong adults. Additionally, understanding dynamic risk factors of sexual offending perpetration may have practical implications in terms of crime prevention and offender rehabilitation. Effective and timely identification and intervention are essential to prevent possible escalation to more serious sexual offending behavior.

Based on the limited empirical studies conducted with community samples in Hong Kong, the lifetime prevalence of sexual victimization (e.g., sexual assault, sexual IPV, and child sexual abuse (CSA)) is estimated to range from less than 1% to 16% [20,21,22,23,24]. In his recent study of 1171 Hong Kong adults aged 18 to 40, Chan [20] found that about 16% (about 11% of men and 19% of women) reported having experienced sexual victimization (after the age of 16). Among these victims, 3% (1% of men and 5% of women) experienced penetrative and 19% (15% of men and 22% of women) experienced nonpenetrative sexual victimization. Turning to offenders, an overall 12% (16% of men and 9% of women) reported engaging in sexual offence perpetration at least once in their lifetime, with 2% as penetrative (2% of men and women) and 6% as nonpenetrative (8% of men and 5% of women) sexual perpetration. Approximately 5% of men and women reported being involved in both sexual offence perpetration and victimization.

Chan and colleagues [22] surveyed a sample of 1154 Chinese adults in Hong Kong who engaged in dating relationships and found that unwanted touch (65%) was the most frequently reported sexually abusive act among male and female victims who had experienced CSA. The lifetime prevalence and preceding-year prevalence of sexual IPV in this sample were estimated to be 9% and 5%, respectively. In another sample of 5049 Hong Kong Chinese adults, Chan [21] estimated the lifetime prevalence of CSA to be 0.9% (0.7% unwanted touch and 0.2% forced sex), while the lifetime prevalence of adult sexual violence (ASV) by nonintimate partners was 0.8% (0.4% unwanted touch, 0.2% forced sex, and 0.2% sexual coercion). Chan [21] also found that women experienced a higher lifetime prevalence of CSA (1.1%) than men (0.6%), but men experienced a higher lifetime prevalence of ASV (0.8%) than women (0.6%).

### 1.1. RSB, Paraphilic Interest, and Sexual Offending Perpetration

#### 1.1.1. RSB and Sexual Offending Perpetration

RSB, such as unprotected vaginal, oral, or anal intercourse, incorrect or inconsistent use of contraceptive measures, and sex with multiple partners and/or high-risk partners (i.e., intravenous drug users), is a global public health concern that impacts many people each year. The Global Burden of Disease Study that includes annual assessments for 188 countries from 1990 to 2013 reported that unsafe sex practiced by young people aged 10 to 24 years was a risk factor for a heightened level of disability-adjusted life-years (the sum of years of potential life lost due to premature death and the years of productive life lost from disability) [25]. According to World Health Organization [26], over a million of people are infected with a sexually transmitted infection (STI) each day. In addition to STIs (including Chlamydia trachomatis (CT), gonorrhea, syphilis, and HIV infections), RSB can result in poor reproductive health outcomes with long-term consequences, such as infertility, unintended pregnancy, and pelvic inflammatory disease [27,28]. In Hong Kong, a population-based geospatial household survey and test (2014–2016) conducted with 881 participants, aged 18 to 49 years, found that the prevalence of CT was low overall (1%) but considerably high (6%) among sexually active young females aged 18 to 26 years [29]. Additionally, global studies (e.g., Addis Ababa, Thailand, and the US) have consistently demonstrated that adolescents and young adults are at an increased risk for adverse sexual health outcomes partly due to their high rates of unprotected sex with multiple partners [30,31,32,33].

As a personality risk factor, an escalation from RSB to sexual offence perpetration (e.g., sexual assault, rape) is frequently reported in the sex offending literature, particularly from the developmental and life-course perspective [34] and the criminal career approach [35]. Furthermore, this notion is consistent with Malamuth et al.’s [36,37] confluence model of sexual aggression. This model is one of the leading explanatory models of sexual aggression and it proposes that “hostile masculinity” is a primary risk factor for males and increases their likelihood to perpetrate sexually aggressive acts. Individuals who have a stronger orientation towards impersonal sex (e.g., having sex earlier in their relationship and sex with partners on only one occasion (“one-night stands”)) and personality traits indicative of hostile masculinity (e.g., misogynistic attitudes) are the most likely to commit sexual violence. Notably, a review of international cross-sectional and longitudinal studies by Davis et al. [38] found that men’s types of sexual partner (e.g., high number of lifetime sexual partners, engage in concurrent or extramarital, have sex with a high-risk sexual partner (e.g., someone who uses intravenous drugs), engage in transactional sex (i.e., the act of exchanging, goods, money, or lifestyle rewards) with women for sex), condom use (e.g., nonconsensual sex without a condom, resistance to or inconsistent condom use), and history of STI diagnosis or symptoms were positively associated with their sexual violence perpetration. In terms of sex differences on RSB, mixed findings were noted. Most studies found that males engaged in significantly more RSB than females [39,40,41,42], although some studies found that females engaged in more RSB than males [43] or no significant sex difference was observed [44].

#### 1.1.2. Paraphilic Interests and Sexual Offending Perpetration

In a broader sense, paraphilias are conditions characterized by persistent atypical sexual interests. The Diagnostic and Statistical Manual of Mental Disorders, Fifth Edition (DSM-5) defined a paraphilia as an “intense and persistent sexual interest other than sexual interest in genital stimulation or preparatory fondling with phenotypically normal, physically mature, consenting human partners” (American Psychiatric Association (APA)) [45]. There are eight paraphilias and their associated paraphilic disorders in the DSM-5, namely voyeurism, exhibitionism, fetishism, transvestic fetishism, frotteurism, sexual sadism, sexual masochism, and pedophilia [46]. Other paraphilias are classified in DSM-5 in the residual diagnosis category of paraphilia (e.g., scatologia, biastophilia, urophilia, scatophiliac, hebephilia, and zoophilia). On the contrary, the International Classification of Diseases, Tenth Revision (ICD-10) identified six disorders or sexual preference, namely voyeurism, exhibitionism, fetishism, fetishistic transvestism, sadomasochism, and pedophilia [47]. In view of the flexibility of sexual norms across time and cultural practices, the DSM-5 distinguished paraphilias from paraphilic disorders, with the former considered as atypical but not inherently pathological behaviors. Paraphilic disorders are regarded as the presence of deviant, maladaptive erotic urges, which may result in a significant threat to the psychological and physical wellbeing of the affected individuals and/or others [45]. On the other hand, paraphilic interest is defined as sexual arousal obtained from an atypical sexual activity (e.g., exposing one’s genitals to nonconsenting others) or target (e.g., prepubescent children) [48]. Nevertheless, it should be noted that having or acting on a paraphilic interest may not necessarily be pathological because most individuals with paraphilic interest do not have a mental disorder. Moreover, paraphilia is only clinically diagnosed if the paraphilic interest is recurrent, persistent, necessary for sexual enjoyment, and results in significant distress or impairment of occupational functioning. Concerning sex differences in paraphilic interests, recent studies with mostly nonclinical samples reported that males typically reported less repulsion (or having more sexual arousal) than females for most types of paraphilic interest [48,49,50]. This is a recent study of paraphilic interests of 1171 Hong Kongers aged 18–40 years. Chan [50] found that males reported significantly higher levels of general and 12 subtypes of paraphilic interest (i.e., voyeurism, exhibitionism, scatologia, fetishism, frotteurism, sadism, biastophilia, urophilia, scatophiliac, hebephilia, pedophilia, and zoophilia) than females, while females had a higher level of transvestic fetishism than males. It was also found that in general, high levels of negative temperament, alcohol and drug use, risky sexual behavior, perceived neighborhood disorganization, and low levels of self-control and social bonds were significant factors associated with the participants’ tendency of having general and 14 subtypes of paraphilic interest. Unlike the present study that explored the role of RSB and paraphilic interests in self-reported sexual offending behavior, Chan’s [50] study examined the psychosocial factors associated with general and subtypes of paraphilic interest in a large group of young male and female adults in Hong Kong.

Recent research has demonstrated that paraphilic interests are positively associated with subsequent involvement in paraphilic activities [51,52,53], which include sexual activities that are inherently illegal (i.e., sexual offending) if they are acted upon nonconsenting individuals (e.g., biastophilia (sexual arousal from sexually assaulting nonconsenting victims), hebephilia (perverse attraction to pubescent children), pedophilia, sadism, and frotteurism). Paraphilic interests are considered a motivational factor for some sexual offenses (e.g., biastophilia, sadism, and pedophilia) [54]. Drury et al. [55] reported that majority of offenders incarcerated for a sexual offense were diagnosed with one or more different paraphilias: pedophilia (57%), paraphilia not otherwise specified (35%), exhibitionism (26%), and voyeurism (21%). These offenders were also likely to have suffered from adverse childhood experiences (e.g., paternal abandonment or neglect, and physical, verbal, emotional, and/or sexual abuse). Sexual sadism and pedophilia were found to be the two most prevalent paraphilias diagnosed among sex offenders [56,57,58,59]. Nevertheless, not all individuals diagnosed with paraphilia have acted upon their sexual interests, many sex offenders are not paraphilic [60,61], and some offenders successfully conceal their paraphilic conditions during diagnostic assessment.

### 1.2. The Present Study

Culture and society are acknowledged as playing an influential role in recognizing and accepting certain behaviors as normal or deviant. According to Reiss [62], sexual practices are often associated with the societal kinship structures and power gradients, which regularly follow prescribed and shared cultural scripts that encourage or discourage some types of sexual interests and behaviors. Cultures and societies, in general, are described as either sex-positive (i.e., emphasizes the pleasurable, gratifying, rewarding, and nonreproductive aspects of sex) or sex-negative (i.e., perceives ejaculation and sexual intercourse as a weakness and sexual asceticism is encouraged) [63]. Compared to Western cultures, Asian, Middle Eastern, and African cultures largely adopt a higher restrictive perception on sexual issues. Sex has generally been a taboo in these cultures [64,65]. Even so, attitudes and values regarding sexual interests, activity, and sexuality can be altered accordingly as societies evolve and adopt new customs [66].

Against this background, this study aims to explore self-reported sexual offending behaviors separated into four categories (i.e., general (all types of sexual offenses), nonpenetrative-only (e.g., sexual molestation, child pornography, online sexual offending), penetrative-only (e.g., vaginal, oral, or anal intercourse, foreign object insertion), and nonpenetrative-plus-penetrative (both nonpenetrative and penetrative) sexual offenses) among young adults in Hong Kong. Hong Kong is a semi-autonomous city (a special administrative region) of the People’s Republic of China (PRC) with approximately 95% of the population of Chinese descent. As a modern Chinese society and a major financial hub in the Asia-Pacific region, Hong Kong was once a British colony for more than 150 years before its return to the PRC on 1 July 1997. Hong Kongers tend to blend their modern Western lifestyle with traditional Chinese cultural values and practices. Traditional Chinese culture can be traced back over 4000 years of history, has been maintained by the same language, includes diverse and longstanding schools of thought (e.g., Confucianism, Taoism, and Buddhism), and has provided the Chinese people with a well-rooted identity. Similar to many former British colonies, the criminal justice system in Hong Kong is based on the British common law system, which emphasizes the rule of law and due process [67]. In view of the lack of information on sexual offending in Hong Kong, this study is important for filling the gap in the literature. It remains unclear if RSB and paraphilic interests are useful in helping to explain sexual offending behavior in a Chinese cultural context. More importantly, findings of this study can inform practice (e.g., preventive and intervention measures) through the identification of significant risk factors for sexual offending perpetration. Timely and effective interventions that focus on these risk factors are essential to reducing the propensity of engaging in sexually offending behaviors. Additionally, examining sex differences can allow for the development of more tailored, gender-responsive preventive measures for sexual offending. Based on the extant literature, the following research hypotheses are proposed.

**Hypothesis 1.**  *There are sex differences in self-reported RSB (i.e., general, penetrative, and nonpenetrative behaviors) and paraphilic interest (i.e., general, voyeurism, exhibitionism, scatologia, fetishism, transvestic fetishism, frotteurism, sadism, masochism, biastophilia, urophilia, scatophilia, hebephilia, pedophilia, and zoophilia), such that male participants are expected to have higher mean levels of self-reported RSB and paraphilic interests than female participants.*

**Hypothesis 2.**  *Self-reported RSB and paraphilic interests are associated with different types of self-reported sexual offending behavior (i.e., nonpenetrative-only, penetrative-only, and nonpenetrative-plus-penetrative sexual offenses) even when controlling for the participants’ demographic characteristics (i.e., sex, age, religiosity, marital status, and education).*

## 2. Methods

### 2.1. Participants and Procedure

The study sample consisted of 1885 participants aged at least 18 years were recruited from all eight public (i.e., government-funded) and three private universities in Hong Kong. The participants were 62% female (*n* = 1175) and 38% male (*n* = 710), with a mean age of 20.83 years (*SD* = 2.4, range = 18–44). Most participants were Hong Kongers (84%), without any religious affiliation (72%), single (64%), and post-secondary-school-educated (55%). Ethical approval was obtained from the first author’s institution. A convenience sampling approach was used and most of the participants (about 55%) were recruited within university compounds (e.g., libraries, reading corners, student cafeterias, and common areas); the remaining participants (about 45%) were recruited in classrooms with prior consent from the instructors. Participants were provided the option to either complete the online (i.e., Qualtrics Survey, about 80%) or paper-and-pen (about 20%) questionnaire. The participants’ informed consent was obtained before the questionnaire was administered. Their participation in the study was completely voluntary, and no monetary incentive was offered. The participants were assured that their anonymous responses would only be used for research purposes. The average time taken to complete the questionnaire was 25 min, the response rate was approximately 90%, and the completion rate was about 85%.

### 2.2. Measures

Self-reported measures were used to explore (a) the participants’ prevalence of general (all types of sexual offenses), nonpenetrative-only, penetrative-only, and nonpenetrative-plus-penetrative sexual offenses; (b) the sex differences in general (combined) and subtypes of RSB and paraphilic interests; and (c) the effects of sexual offending risk factors on nonpenetrative-only and nonpenetrative-plus-penetrative sexual offenses. The questionnaires containing these measures were prepared in both English and Chinese. To accommodate the local Chinese population, the English-written scales were first translated by an experienced and academically qualified English-to-Chinese translator. The Chinese version of these scales was then translated back to English to ensure face validity and compared with the original English version to confirm consistency. A pilot study was performed with 20 participants (10 male and 10 female participants) prior to the data collection, and several Chinese translated items were revised to facilitate easier comprehension.

#### 2.2.1. Self-Reported Sexual Offending Perpetration

To measure the participants’ lifetime experience of engaging in sexual offending behavior, two questions were asked to explore whether they had (a) engaged in penetrative sexual offenses (including vaginal, oral, and, anal penetration, and foreign object insertion), and/or (b) engaged in nonpenetrative sexual offenses (e.g., sexual molestation (touching of the victim’s private parts), masturbation of the victim, child pornography). This measure was dichotomized (0 = *no*, 1 = *yes*). If the participants admitted to having engaged in sexually offending behavior, they were then asked about the type of sexual behavior they performed (i.e., penetrative, nonpenetrative, or both (nonpenetrative-plus-penetrative)). The items in this measure were extracted from the list of questions (3 out of 13 items) found in studies conducted by Baum et al. [46] on sexual harassment and stalking victimization. Sample items asked if the participants during their interactions with victims had “Threatened to use force or harmed her/him to have sexual contact against her/his will,” “Put verbal pressure on her/him to have sexual contact against her/his will,” and “Exploited the fact that she/he was unable to resist (e.g., after she/he had too much alcohol or another drug) to have sexual contact or intercourse against her/his will.” The Cronbach’s α of this measure was 0.95 (males = 0.94, females = 0.95).

#### 2.2.2. RSB

The participants’ level of involvement in RSB over the past six months was measured by the slightly modified version (measured prevalence instead of frequency) of 23-item Sexual Risk Survey [68]. This measure had been used in other similar studies with acceptable interitem consistency [69,70,71,72]. This scale contained two subscales, with 16 items on penetrative and 7 items on nonpenetrative RSB. This measure was dichotomized (0 = *no*, 1 = *yes*) with a total score ranging from 0 to 23. A higher score indicates a greater involvement in RSB. Sample items include “Had anal sex without a condom,” (penetrative RSB), “Had sex with someone you don’t know well or just met” (penetrative RSB), and “Had left a social event with someone you just met” (nonpenetrative RSB). The Cronbach’s α of this overall measure was 0.92 (males = 0.92, females = 0.92), while subscales on penetrative RSB was 0.90 (males = 0.90, females = 0.90) and nonpenetrative RSB was 0.80 (males = 0.80, females = 0.80).

#### 2.2.3. Paraphilic Interests

The 40-item Paraphilias Scale was used to measure the participants’ interest in paraphilic activities [73]. This measure was scored on a seven-point Likert scale (−3 = *very repulsive*, +3 = *very arousing*), with a total score ranging from −120 to +120. A higher score indicated a greater interest in paraphilic activities. Thirty-two items from this scale were used to test 14 subtypes of paraphilic interest, whereas the remaining eight items did not clearly refer to as paraphilic activities (e.g., “You are having sex with an adult woman” and “You are having your feet kissed, fondled, and touched”). These subtypes are:

(1) Voyeurism (sexual arousal involving the observation of an unsuspecting individual who is naked, undressing, or engaging in sexual activity; one item);
(2) Exhibitionism (sexual arousal involving the exposure of one’s genitals to an unsuspecting individual; one item);
(3) Scatologia (sexual arousal involving the making of unsolicited and obscene telephone calls; one item);
(4) Fetishism (sexual arousal involving nonliving objects such as shoes and undergarments; three items);
(5) Transvestic fetishism (sexual arousal involving cross-dressing activities; two items);
(6) Frotteurism (sexual arousal involving activities of touching and rubbing against an unsuspecting individual; one item);
(7) Sadism (sexual arousal involving activities of inflicting harm and humiliation on another individual; six items);
(8) Masochism (sexual arousal involving activities of being humiliated, beaten, bound, or otherwise made to suffer; six items);
(9) Biastophilia (sexual arousal involving having sexual intercourse with a nonconsenting individual; two items);
(10) Urophilia (sexual arousal involving contact with urine; two items);
(11) Scatophilia (sexual arousal involving contact with feces; two items);
(12) Hebephilia (sexual arousal involving having (or not having) sexual intercourse with pubescent children; two items);
(13) Pedophilia (sexual arousal involving having (or not having) sexual intercourse with prepubescent children; two items);
(14) Zoophilia (sexual arousal involving having sexual intercourse with animals; one item).

A higher score denotes a greater interest in the corresponding paraphilic activities. Examples of items are, “You are kissing, fondling, and touching someone’s feet” (fetishism), “You are spanking, beating, or whipping someone” (sadism), “You are pretending to rape someone” (biastophilia), and “You are having sex with a girl below the age of 12” (pedophilia). Of note, two items in this scale were created as control items in the original study, as they were referred to sexual interest in adult males and females [73]. The Cronbach’s α of this measure was 0.97 (males = 0.98, females = 0.97), with the alpha coefficients of all subtypes were above the acceptable level of 0.70.

### 2.3. Data Analytic Strategy

To examine sex differences, independent sample t-tests were performed on different types of RSB (i.e., general (all types of sexual offenses), penetrative, and nonpenetrative behavior), whereas Mann–Whitney U tests were used to test the general and 14 subtypes of paraphilic interest (i.e., voyeurism, exhibitionism, scatologia, fetishism, transvestic fetishism, frotteurism, sadism, masochism, biastophilia, urophilia, scatophilia, hebephilia, pedophilia, and zoophilia) because these are highly skewed variables. Binary logistic regressions were next performed to explore the effects of RSB and paraphilic interests on nonpenetrative-only (1 = nonpenetrative-only, 0 = penetrative and nonpenetrative-plus-penetrative) and nonpenetrative-plus-penetrative sexual offenses (1 = nonpenetrative-plus-penetrative, 0 = nonpenetrative-only and penetrative-only), while controlling for the participants’ demographic characteristics (i.e., sex, age, religiosity, marital status, and education). The participants’ religiosity was assessed by how religious they perceived themselves to be on a six-point Likert scale (1 = *not* at all, 6 = *very strongly*). Binary logistic regressions were not computed on penetrative-only sexual offense given its small subsample size (*n* = 40). Pearson correlations of the tested variables were performed; and no correlation at or above 0.70 was found, indicating no collinearity. Multiple test corrections were not applied to avoid masking significant findings. The significance level was set at 0.05.

### 2.4. Ethical Considerations

This study was approved by the ethical review board of the first author’s university. Participants could end their participation, contact the primary investigator, and/or receive professional counseling at any time. Data were collected anonymously with no personal identifying details recorded.

## 3. Results

### 3.1. Self-Reporting Sexual Offending: Offender and Offense Characteristics

Out of the total sample of 1885 participants, 18% of them reported that they had perpetrated a sexual offense perpetration at least once in their lifetime (see Table 1). Significantly more males (23%) than females (15%) having perpetrated a sexual offense (*χ*^2^ = 21.03, Phi = 0.11, *p* < 0.001). Specifically, 8% of the participants (9% males vs. 7% females) engaged in nonpenetrative-only sexual offending behavior. It is noteworthy that only 40 (2%) participants reported that they had engaged in penetrative-only sexual offending behavior, with significantly more males (3%) than females (2%; *χ*^2^ = 5.23, Phi = 0.05, *p* = 0.031). Finally, 8% of the participants reported they had engaged in nonpenetrative-plus-penetrative sexual offending behavior, and again a significant sex difference was observed (11% males vs. 6% females; *χ*^2^ = 11.06, Phi = 0.08, *p* = 0.001).

Table 2 presents the demographic characteristics of the study sample (*N* = 342). Of the participants, 52% were females and 49% were males. The mean age was 20.92 years (*SD* = 2.05, range = 18–35), and no significant sex difference was found (males: *M* = 19.09, *SD* = 0.94 and females: *M* = 19.12, *SD* = 0.86). A large majority of the participants (82%) were local Hong Kongers, slightly over half (51%) of them were non-single, nearly two thirds (65%) had obtained post-secondary school education, and over three quarters (76%) reported having no religious beliefs.

### 3.2. Mean Differences of Sexual Offending Risk Factors

Table 3 shows the mean differences between the male and female participants’ levels of RSB and paraphilic interests. In general, both male and female participants possessed negative (or repulsive) attitudes toward all types of paraphilic interests. Several significant sex differences in the participants’ paraphilic interests were found. Relative to the female participants, the male participants reported significantly higher levels of interest in voyeurism (*U* = 11,644.50, *Z* = −3.43, *p* = 0.001), frotteurism (*U* = 12,451.50, *Z* = −2.39, *p* = 0.017), biastophilia (*U* = 11,405.50, *Z* = −3.64, *p* < 0.001), scatophilia (*U* = 13,070.00, *Z* = −2.04, *p* = 0.041), and hebephilia (*U* = 12,046.00, *Z* = −3.04, *p* = 0.002). However, the female participants reported a significantly higher level of transvestic fetishism (*U* = 11,384.00, *Z* = −3.65, *p* < 0.001) than the male participants. No significant sex difference was observed on RSB.

### 3.3. Effects of RSB and Paraphilic Interests on Sexual Offending Perpetration

With reference to Table 4 and Table 5, binary logistic regressions were computed to examine the effects of sexual offending risk factors (i.e., RSB and paraphilic interests) on the participants’ self-reported sexual offending behavior (i.e., nonpenetrative-only (1 = nonpenetrative-only, 0 = penetrative and nonpenetrative-plus-penetrative) and nonpenetrative-plus-penetrative sexual assault (1 = nonpenetrative-plus-penetrative, 0 = nonpenetrative-only and penetrative-only)), while controlling for their demographic characteristic (i.e., sex, age, religiosity, marital status, and education). All regression models were significant (Model I: Nagelkerke *R*^2^ = 0.21; Model II: Nagelkerke *R*^2^ = 0.29). Pertaining to nonpenetrative-only sexual offense, the participants who possessed a higher level of RSB (Model I: *B* = −0.18, *SE* = 0.04, *p* < 0.001), more specifically, penetrative RSB (Model II: *B* = −0.29, *SE* = 0.08, *p* < 0.001); and paraphilic interests in voyeurism (Model II: *B* = −0.26, *SE* = 0.13, *p* = 0.049) and zoophilia (Model II: *B* = −0.61, *SE* = 0.20, *p* = 0.003) were less likely to engage in nonpenetrative-only sexual offense (see Table 4). Furthermore, being younger (Model I: *B* = −0.18, *SE* = 0.08, *p* = 0.026) and single (Model I: *B* = 0.69, *SE* = 0.25, *p* = 0.006; Model II: *B* = 0.64, *SE* = 0.27, *p* = 0.017) increased the odds of committing nonpenetrative-only sexual offense.

In Table 5, all regression models were significant (Model I: Nagelkerke *R*^2^ = 0.17; Model II: Nagelkerke *R*^2^ = 0.24). The participants who possessed a higher level of RSB (Model I: *B* = 0.12, *SE* = 0.03, *p* < 0.001), and more specifically, penetrative RSB (Model II: *B* = 0.24, *SE* = 0.06, *p* < 0.001), were more likely to engage in nonpenetrative-plus-penetrative sexual offenses. Additionally, the participants who had higher level of paraphilic interests in exhibitionism (Model II: *B* = 0.30, *SE* = 0.17, *p* = 0.049) and zoophilia (Model II: *B* = 0.38, *SE* = 0.18, *p* = 0.037) were more likely to perpetrate both nonpenetrative and penetrative sexual offenses. Being older (Model I: *B* = 0.13, *SE* = 0.07, *p* = 0.049) and not single (Model I: *B* = −0.70, *SE* = 0.24, *p* = 0.004; Model II: *B* = −0.70, *SE* = 0.26, *p* = 0.007) increased the odds of committing both nonpenetrative and penetrative sexual offenses.

## 4. Discussion

This study has offered an initial insight into personality and psychopathological risk factors of sexual offending perpetration in a Hong Kong sample. In addition to advancing our knowledge on sexual offence perpetration, this study is important for its focus on an under-researched population, i.e., Hong Kong adults. In addition to exploring the prevalence of self-reported sexual behaviors (i.e., general, nonpenetrative-only, penetrative-only, and nonpenetrative-plus-penetrative behaviors), this study had two primary aims: (1) to investigate sex differences for RSB (i.e., general, penetrative, and nonpenetrative) and paraphilic interests (i.e., general and 14 subtypes) (Hypothesis 1) and (2) to examine whether the relationship between different types of sexual offending behaviors (i.e., nonpenetrative-only and nonpenetrative-plus-penetrative sexual offenses) and personality (i.e., RSB) psychopathological (i.e., paraphilic interests) risk factors hold when controlling for demographic characteristics (i.e., sex, age, religiosity, marital status, and education) (Hypothesis 2).

In this study, the lifetime prevalence of general sexual offense was 18%, with a significantly higher rate reported in males than in females (23% vs. 15%). Higher rates were also observed in males than in females on different sexual offending behaviors: 8.10% of nonpenetrative-only (9% males vs. 7% females), 2% of penetrative-only (3% males vs. 2% females), and 8% nonpenetrative-plus-penetrative (11% males vs. 6% females) sexual offenses. Consistent with the literature where most studies were conducted in the West, the higher prevalence of sexual offending in males than in females was also observed in this study [13,14]. Interestingly, females reported to have engaged in sexual offending behaviors were composed of 15% of all female participants in this study, which was much higher than commonly reported in the literature (i.e., about 5% based on official reports, clinical data, and victim surveys in the West (e.g., the US, Canada, the UK, Australia, New Zealand)) [14,74]. Nonetheless, Denov [75] reported that though official data in the West suggested female offending prevalence rates between 2% and 6%, self-report data suggested a prevalence rate up to 58%. Hence, a high prevalence rate of female sexual offending reported in a community survey was not uncommon.

Overall, in the present study, male participants reported significantly more paraphilic interests in voyeurism, frotteurism, biastophilia, scatophilia, and hebephilia than the female participants, while the female participants reported significantly more transvestic fetishism than their male counterparts. Therefore, the male participants were generally found to possesses a higher tendency of paraphilic interests than the female participants, which is consistent with many studies conducted with Western samples [76,77,78]. For instance, Joyal and Carpentier [77] found in their 1040 adults (475 men and 565 women) that significantly more men than women reported paraphilic interests in voyeurism (60% vs. 35%) and frotteurism (34% vs. 21%), while significantly more women than men reported interests in masochism (28% vs. 19%).

There were several noteworthy findings with respect to the role of RSB and paraphilic interest in committing sexual offenses that warrant further discussion. In general, the participants’ RSB was a significant risk factor of sexually offending behavior. RSB, specifically penetrative behavior, decreased the odds of the participants’ propensity to engage in nonpenetrative sexual assault. The opposite was true for the participants’ tendency to engage in both nonpenetrative and penetrative sexual assault, with RSB (specifically penetrative behavior) being a positive risk factor. In other words, those who engaged in penetrative RSB were less likely to commit only nonpenetrative sexual assault. Of note, earlier research on university-based sexual offending has supported the role of deviant and risky sexual fantasies as a precursor to sexual offending [79,80]. Consistently, sexual risk-taking behavior was found to be a significant predictor of subsequent sexual offending behavior in more recent studies [34,35,39]. Of note, it is possible that individuals who had committed sexual offending behavior also have a higher tendency to subsequently engage in RSB and to possess paraphilic interests, potentially leading to sexual recidivism [81,82]. Although general paraphilic interest was not found to be significantly associated with any type of sexual assault (the insignificant relationship between general paraphilic interests and different types of sexual assault was possibly due to the overall reported low interest levels of most paraphilic interest subtypes), a significant relationship was noted in several specific paraphilic interests. The participants’ interest in zoophilic activities reduced the odds of their tendency to engage on nonpenetrative-only sexual assault but increased the odds of their propensity to commit both nonpenetrative and penetrative sexual assault. In addition, possessing a paraphilic interest in voyeurism lowered the odds of the participants to engage in nonpenetrative-only sexual assault, while the participants’ interest in exhibitionism was a significant risk factor of their tendency to engage in both nonpenetrative and penetrative sexual assault. Paraphilic interests and behaviors are found to be positively correlated [54]. Hence, it is plausible that those who possess interest in paraphilic behaviors may subsequently act on them. These findings are in line with the extant literature whereby paraphilic interests and diagnoses of paraphilia are commonly observed in sex offenders. Studies have found a high prevalence of paraphilias (58% to 98%) among those who engaged in sexually offending behavior [78,80]. These paraphilic interests, and oftentimes paraphilic activities, can range from a sexual preference for children to sadism to nonconsenting coercive sex. In fact, Carvalho [83] and Chan [20] posited that paraphilic interest can escalate to becoming a motivational factor in some sexual offenses.

In terms of the participants’ demographic characteristics, the findings demonstrate that the younger participants were more likely to engage in nonpenetrative sexual offense, while the older participants were in a higher tendency to commit both types of nonpenetrative and penetrative sexual offenses. Studies have demonstrated that escalation in severity of sexual offending (i.e., from nonpenetrative (e.g., child molestation, online sex offending) to penetrative (rape and other contact-based sexual assault) sexual offending) is not unusual among sex offenders [84,85,86]. Sex offenders who commit nonpenetrative and penetrative sexual offenses are commonly referred to as “dual offenders” or “mixed offenders” [87]. According to Chan et al.’s [88] social learning–routine activity integrated theory, when the mere indulgence in deviant and paraphilic sexual fantasies for sexual pleasure and excitement no longer is sufficient to produce sufficient sexual euphoria, the individuals may then act out their deviant and paraphilic sexual fantasies to restore a more desirable level of sexual gratification [89]. Hence, escalation in severity from minor sexual offending (e.g., sexual molestation) to more serious sexual penetration (e.g., sexual assault, rape, sexual murder) is one pathway for how sexual offending can evolve over time.

In addition, participants who reported being single were found to have a higher odds than their non-single (i.e., in a relationship) counterparts to engage in nonpenetrative sexual offense. Although research on sex offender’s marital status has shown mixed results, some studies reported that more than 50% of child pornography offenders (i.e., nonpenetrative sex offenders) were single [90,91,92]. Van Wijk et al. [93] postulated that these nonpenetrative sex offenders (e.g., child pornography offenders) often have marital (or intimate relationship) problems and power imbalance (or perception of gender inequality) between intimate partners. However, the opposite was true for non-single offenders who were more likely than their single counterparts to commit nonpenetrative and penetrative sexual offenses. Additionally, studies comparing homicidal with nonhomicidal sex offenders found that a large majority of nonhomicidal sex offenders (73% to 88%) were involved in a sexual relationship at the time of their offense [94,95,96].

The findings of this study should be interpreted cautiously in view of a number of limitations. First, this study was limited to self-reported data, and this affected the depth of participants’ responses concerning their sexually offending behavior. In addition, the participants were not surveyed for the number of sexual offending incidents they had engaged in during their lifetime; hence, this measure was unable to estimate the intensity and severity of their reported sexual offending behavior. Moreover, biases such as social desirability and retrospective recall bias may have influenced the participants’ truthfulness in reporting their sexual interests (e.g., paraphilic interests), practices (e.g., RSB), and offending behaviors (e.g., nonpenetrative and penetrative sexual offenses). This can be relevant to the generally low interest in most paraphilic interests in this study. Participants largely demonstrated lesser interest (or high repulsiveness) in most paraphilic interests (e.g., sadism, masochism). Furthermore, some of the Western-developed measures used in this study have not been culturally validated, so the validity of these measures used in an Asian sample remains unclear. Therefore, future research could incorporate a measure for response bias to minimize participants’ potential reporting biases, use more culturally specific measures as available, and explore additional offense-related factors, such as victim characteristics (e.g., victim–offender relationship) and other offender and offense characteristics (e.g., the offender’s motivation, presence of personality disorders or other psychiatric diagnoses, and situational influences) to better understand this type of sexual offender population (i.e., university-based self-reported sexual offenders). Next, given the cross-sectional nature of this study, it failed to examine the causal relationships between the participants’ risk factors and their self-reported sexual offending behaviors. Therefore, future research should consider adopting a longitudinal framework to obtain a better understanding of the sexual offending phenomenon in this population. Finally, this study only recruited participants from universities, and hence, the findings cannot be generalized to a wider population. Of note, the small sample size in this study may reduce the statistical power and effect of the findings. Hence, future research should recruit a larger sample size and participants from all walks of life in Hong Kong.

## 5. Conclusions

This study is important for filling the gap in the literature and provides a solid groundwork for further research. Notwithstanding its limitations, several major implications derived from the findings can be offered. Notably, RSB (e.g., unprotective sex, multiple sexual partners) and deviant paraphilic interests (e.g., nonconsenting coercive sex, sadistic activities, and a sexual preference for children) were found to be important factors influencing the participants’ involvement in nonpenetrative and penetrative sexual assault. Therefore, it remains imperative to raise public awareness about the adverse consequences of RSB and deviant paraphilic interests, the importance of a healthy sexual lifestyle, and more importantly, the potential for escalation to the actual commission of sexual offenses (e.g., nonconsenting sexual molestation and penetration). This public education effort should occur as soon in life as developmentally appropriate. Relevant to this study, school-based sex education has long been criticized for not being comprehensive enough [97]. It is not surprising that topics related to sex are often a cultural taboo in many traditional Asian societies. School- and university-based public awareness campaigns should also include educational material on the risks associated with the premature onset of sexual activity and the use of alcohol and other drugs. Reviews have demonstrated that alcohol and substance (mis)use are found to be associated with sexual assault perpetration [98,99]. Additionally, relevant behavioral changes, such as shaping attitudes toward safer and more socially acceptable sexual practices (e.g., nonparaphilic activities), the importance of condom use for protected sex, limiting the number of sexual partners, and avoiding alcohol consumption and drug use before sex are important educational messages, especially for the younger population. Understanding of other sexuality aspects, such as general sexual inhibition and excitation, are also important, as these may relate to the development of deviant paraphilic interests [48]. Nonetheless, approaches to sex education in Hong Kong and other Chinese societies, such as mainland China, Macau, Taiwan, and Singapore, should be cautiously planned with cultural sensitivity in mind, as Chinese culture is traditionally highly conservative on sexual attitudes and practices. Other community-based preventive efforts, such as public mental health seminars, can be regularly organized to underscore the importance of only engaging in sexual activity with appropriate and consenting partners. This helps promote healthy intimate relationships, an increased sense of closeness, and minimizes loneliness. Such educational efforts have also been found to be a protective factor against sexual offending behavior [100].

In terms of offender rehabilitation, social norm interventions with treatment targeting the individual’s sexual misperceptions and sensation-seeking should be provided for those who are identified as being at high risk of RSB or who have deviant paraphilic interests or behaviors of concern [50,101]. Research has consistently demonstrated that sexual sensation-seeking (e.g., engaging in RSB and paraphilic activities) is positively correlated with subsequent sexual offending behavior [102,103]. These interventions are likely to have both a remedial effect by reducing the frequency with individuals who already engage in a behavior (e.g., RSB, paraphilic interests), and a preventive effect by correcting misperceptions among those who do not or only rarely engage in such behavior. It is noteworthy that any prevention and intervention strategies should be culturally sensitive in order to achieve the optimal effect.

## Figures and Tables

**Table 1:** Lifetime prevalence and sex differences of sexual offending perpetration (*N* = 1885).

Sexual Offending Behavior	All Sample (*N* = 1885)		Male (*n* = 710)		Female (*n* = 1175)		Sex Differences	
	*N*	Percent	*n*	Percent	*n*	Percent	*χ* ^2^	Phi
General sexual assault	342	18.1%	166	23.4%	176	15%	21.03	0.11 ***
Nonpenetrative behavior only	153	8.1%	67	9.4%	86	7.3%	2.66	0.04
Penetrative behavior only	40	2.1%	22	3.1%	18	1.5%	5.23	0.05 *
Nonpenetrative-plus-penetrative behavior	149	7.9%	75	10.6%	74	6.3%	11.06	0.08 **

* *p* < 0.05, ** *p* < 0.01, *** *p* < 0.001.

**Table 2:** Demographic characteristics of the self-reported sexual offenders (*N* = 342).

Variable	*N*	Percentage
Sex		
Male	166	48.5%
Female	176	51.5%
Country of origin		
Hong Kong	280	81.9%
Mainland China	50	14.6%
Others (e.g., Macau, South Korea, Canada, USA, Taiwan, Thailand, the Netherlands, and Serbia)	12	3.5%
Marital status		
Single	168	49.1%
Non-single	171	50.9%
Highest education attainment		
Secondary school education	121	35.4%
Post-secondary school education (e.g., associate degree/high diploma; and undergraduate and postgraduate degrees)	221	64.6%
Religious belief		
Without a religious belief	261	76.3%
With a religious belief (e.g., Christianity, Catholic, Buddhism, and Muslim)	81	23.7%

**Table 3:** Mean level differences of sexual offending risk factors.

**Risk Factors**	**All Sample** **(*N* = 342)**		**Male** **(*n* = 166)**		**Female** **(*n* = 176)**		
	***M***	***SD***	***M***	***SD***	***M***	***SD***	***t***
Risky sexual behavior							
General behavior	3.11	4.55	3.26	4.57	2.96	4.53	−0.61
Penetrative behavior	1.95	3.21	2.05	3.25	1.85	3.17	−0.60
Nonpenetrative behavior	1.16	1.71	1.20	1.72	1.11	1.70	−0.49
	***M***	***SD***	**Mean Rank**		**Mean Rank**		***Z* Value**
Paraphilic interests							
General interest	−68.65	39.70	174.06		169.09		−0.46
Voyeurism	−1.63	1.68	188.35		154.54		−3.43 **
Exhibitionism	−2.14	1.37	174.84		167.35		−0.82
Scatologia	−2.26	1.29	170.82		171.17		−0.04
Fetishism	−2.13	2.98	173.80		169.33		−0.42
Tranvestic Fetishism	−3.22	2.75	152.08		189.82		−3.65 ***
Frotteurism	−1.77	1.53	182.54		159.15		−2.39 *
Sadism	−9.58	7.55	171.28		171.70		−0.04
Masochism	−9.90	7.15	161.70		180.75		−1.79
Biastophilia	−3.57	2.92	189.79		153.17		−3.64 ***
Urophilia	−4.65	2.27	180.63		162.89		−1.93
Scatophilia	−4.86	2.29	179.77		162.69		−2.04 *
Hebephilia	−4.27	2.44	186.93		156.94		−3.04 **
Pedophilia	−4.57	2.30	177.37		165.96		−1.22
Zoophilia	−2.31	1.31	173.97		168.18		−0.71

* *p* < 0.05, ** *p* < 0.01, *** *p* < 0.001.

**Table 4:** Binary logistic regression models of self-reported nonpenetrative-only sexual assault (*n* = 153).

Risk Factors	Model I		Model II	
	*b* (*SE*)	OR (CI)	*b* (*SE*)	OR (CI)
Demographic characteristics				
Sex (0 = female, 1 = male)	−0.33 (0.25)	0.72 (0.45, 1.17)	−0.42 (0.29)	0.66 (0.37, 1.16)
Age	−0.18 (0.08)	0.84 (0.72, 0.98) *	−0.13 (0.09)	0.88 (0.74, 1.05)
Religiosity	−0.09 (0.07)	0.92 (0.80, 1.05)	−0.12 (0.08)	0.89 (0.76, 1.04)
Marital status (0 = non-single, 1 = single)	0.69 (0.25)	1.98 (1.22, 3.22) **	0.64 (0.27)	1.89 (1.12, 3.19) *
Education (0 = secondary, 1 = post-secondary)	−0.03 (0.28)	0.97 (0.56, 1.68)	−0.07 (0.30)	0.93 (0.52, 1.67)
Sexual offending risk factors				
Risky sexual behaviors	−0.18 (0.04)	0.84 (0.77, 0.91) ***		
Nonpenetrative behavior			0.03 (0.12)	1.03 (0.82, 1.30)
Penetrative behavior			−0.29 (0.08)	0.75 (0.65, 0.87) ***
Paraphilic interests	0.01 (0.01)	1.00 (1.00, 1.01)		
Voyeurism			−0.26 (0.13)	0.77 (0.59, 1.00) *
Exhibitionism			−0.07 (0.18)	0.93 (0.66, 1.32)
Scatologia			0.08 (0.20)	1.08 (0.73, 1.61)
Fetishism			0.09 (0.06)	1.10 (0.98, 1.22)
Tranvestic fetishism			0.01 (0.07)	1.00 (0.87, 1.15)
Frotteurism			0.15 (0.13)	1.16 (0.90, 1.50)
Sadism			0.03 (0.04)	1.03 (0.95, 1.12)
Masochism			0.01 (0.04)	1.00 (0.92, 1.08)
Biastophilia			−0.02 (0.08)	0.98 (0.84, 1.16)
Urophilia			0.15 (0.12)	1.17 (0.92, 1.48)
Scatophilia			−0.15 (0.13)	0.86 (0.67, 1.11)
Hebephilia			0.24 (0.14)	1.27 (0.95, 1.68)
Pedophilia			0.03 (0.13)	1.04 (0.80, 1.34)
Zoophilia			−0.61 (0.20)	0.54 (0.36, 0.81) **
Constant	4.32 (1.63)	74.90 **	3.09 (1.81)	21.95 *
Model *χ*^2^	57.37 ***		80.91 ***	
Nagelkerke *R*^2^	0.21		0.29	
Hosmer–Lemeshow test	26.35		6.81	

Notes: unstandardized beta (*b*) and standard error (SE). * *p* < 0.05, ** *p* < 0.01, *** *p* < 0.001.

**Table 5:** Binary logistic regression models of self-reported nonpenetrative-plus-penetrative sexual assault (*n* = 149).

Risk Factors	Model I		Model II	
	*b* (*SE*)	OR (CI)	*b* (*SE*)	OR (CI)
Demographic characteristics				
Sex (0 = female, 1 = male)	0.06 (0.24)	1.06 (0.66, 1.71)	0.12 (0.28)	1.13 (0.65, 1.98)
Age	0.13 (0.07)	1.14 (0.99, 1.31) *	0.06 (0.07)	1.07 (0.92, 1.23)
Religiosity	0.01 (0.07)	1.00 (0.99, 1.31)	0.06 (0.08)	1.06 (0.91, 1.22)
Marital status (0 = non-single, 1 = single)	−0.70 (0.24)	0.50 (0.31, 0.80) **	−0.70 (0.26)	0.49 (0.30, 0.82) **
Education (0 = secondary, 1 = post-secondary)	0.40 (0.28)	1.49 (0.87, 2.55)	0.46 (0.29)	1.59 (0.90, 2.80)
Sexual offending risk factors				
Risky sexual behaviors	0.12 (0.03)	1.13 (1.06, 1.21) ***		
Nonpenetrative behavior			−0.10 (0.11)	0.91 (0.73, 1.13)
Penetrative behavior			0.24 (0.06)	1.27 (1.12, 1.44) ***
Paraphilic interests	0.01 (0.01)	1.00 (0.99, 1.00)		
Voyeurism			0.05 (0.13)	1.05 (0.82, 1.34)
Exhibitionism			0.30 (0.17)	1.35 (0.97, 1.87) *
Scatologia			−0.08 (0.18)	0.92 (0.65, 1.31)
Fetishism			−0.08 (0.05)	0.92 (0.83, 1.03)
Tranvestic fetishism			−0.05 (0.07)	0.95 (0.83, 1.09)
Frotteurism			0.01 (0.13)	1.00 (0.78, 1.28)
Sadism			0.03 (0.04)	1.03 (0.95, 1.11)
Masochism			−0.06 (0.04)	0.95 (0.87, 1.02)
Biastophilia			−0.08 (0.08)	0.92 (0.79, 1.08)
Urophilia			−0.01 (0.12)	0.99 (0.78, 1.25)
Scatophilia			0.01 (0.12)	1.00 (0.79, 1.28)
Hebephilia			−0.22 (0.14)	0.81 (0.61, 1.06)
Pedophilia			0.12 (0.13)	1.13 (0.87, 1.45)
Zoophilia			0.38 (0.18)	1.46 (1.02, 2.10) *
Constant	−3.58 (1.49)	0.03 **	−2.15 (1.55)	0.12 *
Model χ^2^	47.15 ***		65.46 ***	
Nagelkerke *R*^2^	0.17		0.24	
Hosmer–Lemeshow test	19.81		9.89	

Notes: unstandardized beta (*b*) and standard error (SE). * *p* < 0.05, ** *p* < 0.01, *** *p* < 0.001.

## Data Availability

The data presented in this study are available on request from the corresponding author.

## References

[1] Chan H.C.O. (2023). Sexual Offending in Asia: A Psycho-Criminological Perspective.

[2] Seto M.C., Kingston D.A., Stephens S., Cutler B.L., Zapf P.A. (2015). Sexual offending. APA Handbook of Forensic Psychology, Vol. 1. Individual and Situational Influences in Criminal and Civil Contexts.

[3] Camilleri J.A., Shackelford T.K., Weekes-Shackelford V.A. (2012). Evolutionary psychological perspectives on sexual offending: From etiology to intervention. Oxford Handbook of Evolutionary Perspectives on Violence, Homicide, and War.

[4] Adjorlolo S., Chan H.C.O. (2017). The nature of instrumentality and expressiveness of homicide crime scene behaviors: A review. Trauma Violence Abus..

[5] Chan H.C.O. (2019). A Global Casebook of Sexual Homicide.

[6] Williams K.S., Bierie D.M. (2015). An incident-based comparison of female and male sexual offenders. Sex Abus..

[7] Chan H.C.O., Heide K.M. (2008). Weapons used by juveniles and adult offenders in sexual homicides: An empirical analysis of 29 years of U.S. data. J. Investig. Psychol. Offender Profiling.

[8] Harris D.A., Ackerman A., Haley M. (2017). ‘Losing my religion:’ An exploration of religion and spirituality in men who claim to have desisted from sexual offending. Crim. Justice Stud..

[9] Navarro J.N., Jasinski J.L. (2015). Demographic and motivation differences among online sex offenders by type of offense: An exploration of routine activities theories. J. Child Sex. Abus..

[10] Faust E., Bickart W., Renaud C., Camp S. (2015). Child pornography possessors and child contact sex offenders: A multilevel comparison of demographic characteristics and rates of recidivism. Sex. Abus..

[11] Garcia-Moreno C., Jansen H.A.F.M., Ellsberg M., Heise L., Watts C.H., on behalf of the WHO Multi-country Study on Women’s Health and Domestic Violence against Women Study Team (2006). Prevalence of intimate partner violence: Findings from the WHO multi-country study on women’s health and domestic violence. Lancet.

[12] World Health Organization (2013). Global and Regional Estimates of Violence against Women: Prevalence and Health Effects of Intimate Partner Violence and Non-Partner Sexual Violence.

[13] Piquero A., Farrington D.P., Jennings W., Diamond B., Craig J. (2012). Sex offenders and sex offending in the Cambridge study in delinquent development: Prevalence, frequency, (dis)continuity over the life-course. J. Crime Justice.

[14] Cortoni F., Hanson R.K. (2005). A Review of the Recidivism Rates of Adult Female Sexual Offenders. Correctional Services of Canada. https://www.csc-scc.gc.ca/research/092/r169_e.pdf.

[15] United Nations Office on Drugs and Crime (2017). Statistics on Crime: Sexual Violence. https://dataunodc.un.org/data/crime/sexual-violence.

[16] Hong Kong Police Force (2022). Hong Kong Crime Statistics. https://www.police.gov.hk/ppp_en/09_statistics/index.html.

[17] Carpentier J., Proulx J. (2011). Correlates and recidivism among adolescents who have sexually offended. Sex. Abus..

[18] Riser D.K., Pegram S.E., Farley J.P. (2013). Adolescent and young adult male sex offenders: Understanding the role of recidivism. J. Child Sex. Abus..

[19] Tharp A.T., Valle L.A., Brookmayer K.A., Massetti G.M., Matjasko J.L. (2013). A systematic qualitative review of risk and protective factors for sexual violence perpetration. Trauma Violence Abus..

[20] Chan H.C.O. (2021). The victim-offender overlap in sexual offending: Exploring a community-based sample of young adults in Hong Kong. Sex. Abus..

[21] Chan K.L. (2011). Association between childhood sexual abuse and adult sexual victimization in a representative sample in Hong Kong Chinese. Child Abus. Negl..

[22] Chan K.L., Yan E., Brownridge D.A., Tiwari A., Fong D.Y.T. (2011). Childhood sexual abuse associated with dating partner violence and suicidal ideation in a representative household sample in Hong Kong. J. Interpers. Violence.

[23] Lau Y., Chan K.S. (2007). Influence of intimate partner violence during pregnancy and early postpartum depressive symptoms on breastfeeding among Chinese women in Hong Kong. J. Midwifery Women’s Health.

[24] Tang C.S. (2002). Childhood experience of sexual abuse among Hong Kong Chinese college students. Child Abus. Negl..

[25] Mokdad A.H., Forouzanfar M.H., Daoud F., Mokdad A.A., El Bcheraoui C., Moradi-Lakeh M., Kyu H.H., Barber R.M., Wagner J., Cercy K. (2016). Global burden of diseases, injuries, and risk factors for young people’s health during 1990–2013: A systematic analysis for the Global Burden of Disease Study 2013. Lancet.

[26] World Health Organization Sexually Transmitted Infections (STIs). https://www.who.int/en/news-room/fact-sheets/detail/sexually-transmitted-infections-(stis).

[27] Abajobir A.A., Kisely S., Maravilla J.C., Williams G., Najman J.M. (2017). Gender differences in the association between childhood sexual abuse and risky sexual behaviours: A systematic review and meta-analysis. Child Abus. Neglect..

[28] Hoyle R.H., Fejfar M.C., Miller J.D. (2000). Personality and sexual risk taking: A quantitative review. J. Personal..

[29] Wong W.C.W., Zhao Y., Wong N.S., Parish W.L., Miu H.Y.H., Yang L., Emch M., Ho K.M., Fong F.Y., Tucker J.D. (2017). Prevalence and risk factors of chlamydia infection in Hong Kong: A population-based geospatial household survey and testing. PLoS ONE.

[30] DiClemente R., Milhausen R.R., Salazar L.F., Spitalnick J., Sales J.M., Crosby R.A., Younge S.N., Wingood G.M. (2010). Development of the sexual sensation-seeking scale for African American adolescent women. Int. J. Sex. Health.

[31] Fetene N., Mekonnen W. (2018). The prevalence of risky sexual behaviors among youth center reproductive health clinics users and non-users in Addis Ababa, Ethiopia: A comparative cross-sectional study. PLoS ONE.

[32] Martinez G., Copen C.E., Abma J.C. Teenagers in the United States: Sexual Activity, Contraceptive Use, and Childbearing, 2006–2010 National Survey of Family Growth. Vital and Health Statistics (Series 23, Number 31). https://www.cdc.gov/nchs/data/series/sr_23/sr23_031.pdf.

[33] Pinyopornpanish K., Thanamee S., Jiraporncharoen W., Thaikla K., McDonald J., Aramrattana A., Angkurawaranon (2017). Sexual health, risky sexual behavior and condom use among adolescents, young adults, and older adults in Chiang Mai, Thailand: Findings from a population based survey. BMC Res. Notes.

[34] Smallbone S., Cale J., Blockland A., Lussier P. (2015). An integrated life-course developmental theory of sexual offending. Sex Offenders: A Criminal Career Approach.

[35] Lussier P., Cale J. (2013). Beyond sexual recidivism: A review of the sexual criminal career parameters of adult sex offenders. Aggress. Violent Behav..

[36] Malamuth N.M., Sockloskie R.J., Koss M.P., Tanaka J.S. (1991). Characteristics of aggressors against women: Testing a model using a national sample of college students. J. Consult. Clin. Psychol..

[37] Malamuth N.M., Linz D., Heavey C.L., Barnes G., Acker M. (1995). Using the confluence model of sexual aggression to predict men’s conflict with women: A 10-year follow-up study. J. Personal. Soc. Psychol..

[38] Davis K.C., Neilson E.C., Wegner R., Danube C.L. (2018). The intersection of men’s sexual violence perpetration and sexual risk behavior: A literature review. Aggress. Violent Behav..

[39] Chan H.C.O. (2021). Risky sexual behavior of young adults in Hong Kong: An exploratory study of psychosocial risk factors. Front. Psychol..

[40] Bongers I.L., Koot H.M., Van der Ende J., Verhulst F.C. (2003). The normative development of child and adolescent problem behavior. J. Abnorm. Psychol..

[41] Kotchick B.A., Shaffer A., Miller K.S., Forehand R. (2001). Adolescent sexual risk behavior: A multi-system perspective. Clin. Psychol. Rev..

[42] Newman P.A., Zimmerman M.A. (2000). Gender differences in HIV-related sexual risk behavior among urban African American youth: A multivariate approach. AIDS Educ. Prev..

[43] Murphy D., Rotheram-Borus M.J., Reid H. (1998). Adolescent gender differences in HIV-related sexual risk acts, social-cognitive factors and behavioral skills. J. Adolesc..

[44] Crockett L.J., Raffaelli M., Shen Y. (2006). Linking self-regulation and risk proneness to risky sexual behavior: Pathways through peer pressure and early substance use. J. Res. Adolesc..

[45] American Psychiatric Association (2013). Diagnostic and Statistical Manual of Mental Disorders.

[46] Baum K., Catalano S., Rand M., Rose K. (2009). Stalking Victimization in the United States.

[47] World Health Organization International Statistical Classification of Diseases and Related Health Problems (10th rev.). https://icd.who.int/browse10/2010/en.

[48] Dawson S.J., Bannerman B.A., Lalumière M.L. (2016). Paraphilic interests: An examination of sex differences in a nonclinical sample. Sex. Abus..

[49] Bouchard K.N., Dawson S.J., Lalumière M.L. (2017). The effects of sex drive and paraphilic interests on paraphilic behaviours in a nonclinical sample of men and women. Can. J. Hum. Sex..

[50] Chan H.C.O. (2022). Paraphilic interests: The role of psychosocial factors in a sample of young adults in Hong Kong. Sex. Res. Soc. Policy.

[51] Levaque E., Dawson S.J., Wan C., Lalumière M.L. (2022). Sex drive as a possible mediator of the gender difference in the prevalence of paraphilic interests in a nonclinical sample. Arch. Sex. Behav..

[52] Bártová K., Androvičová R., Krejčová L., Weiss P., Klapilová K. (2021). The prevalence of paraphilic interests in the Czech population: Preference, arousal the use of pornography, fantasy, and behavior. J. Sex Res..

[53] Joyal C.C., Carpentier J. (2022). Concordance and discordance between paraphilic interests and behaviors: A follow-up study. J. Sex Res..

[54] Seto M.C., Curry S., Dawson S.J., Bradford J.M., Chivers M.L. (2021). Concordance of paraphilic interests and behaviors. J. Sex Res..

[55] Drury A., Heinrichs T., Elbert M., Tahja K., DeLisi M., Caropreso D. (2017). Adverse childhood experiences, paraphilias, and serious criminal violence among federal sex offenders. J. Crim. Psychol..

[56] Chan H.C.O., Beauregard E., Myers W.C. (2015). Single-victim and serial sexual homicide offenders: Differences in crime, paraphilias, and personality traits. Crim. Behav. Ment. Health.

[57] Jackson R.L., Richards H.J. (2007). Diagnostic and risk profiles among civilly committed sex offenders in Washington State. Int. J. Offender Ther. Comp. Criminol..

[58] McElroy S.L., Soutullo C.A., Taylor P., Nelson E.B., Beckman D.A., Brunsman L.A., Ombaba J.M., Keck P.E. (1999). Psychiatric features of 36 men convicted of sexual offenses. J. Clin. Psychiat..

[59] Myers W.C., Chan H.C.O., Vo E.J., Lazarou E. (2010). Sexual sadism, psychopathy, and recidivism in juvenile sexual murderers. J. Investig. Psychol. Offender Profiling.

[60] Cantor J.M., McPhail I.V. (2016). Non-offending pedophiles. Current Sex. Health Rep..

[61] Seto M.C. (2013). Internet Sex Offenders.

[62] Reiss I.L. (1986). Journey into Sexuality: An Exploratory Voyage.

[63] Bullough V.L. (1976). Sexual Variance in Society and History.

[64] Baazeem A. (2016). Challenges to practicing sexual medicine in the Middle East. Sex. Med. Rev..

[65] Ho C.C., Singam P., Hong G.E., Zainuddin Z.M. (2011). Male sexual dysfunction in Asia. Asian J. Androl..

[66] Ayonrinde O., Bhugra D., Bhugra D., Malhi G.S. (2015). Paraphilias and culture. Troublesome Disguises: Managing Challenging Disorders in Psychiatry.

[67] Chan H.C.O., Jennings W.G., Higgins G.E., Khey D.N., Maldonado-Molina M.M. (2016). Crime and punishment in Hong Kong. The Encyclopedia of Crime and Punishment.

[68] Turchik J.A., Garske J.P. (2009). Measurement of sexual risk taking among college students. Arch. Sex. Behav..

[69] Birthrong A., Latzman R.D. (2014). Aspects of impulsivity are differentially associated with risky sexual behaviors. Pers. Individ. Differ..

[70] Fulton J.J., Marcus D.K., Payne K.T. (2010). Psychopathic personality traits and risky sexual behavior in college students. Pers. Individ. Differ..

[71] Lemley S.M., Fleming W.A., Jarmolowicz D.P. (2017). Behavioral economic predictors of alcohol and sexual risk behavior in college drinkers. Psychol. Record.

[72] Walsh K., Latzman N.E., Latzman R.D. (2014). from child sexual and physical abuse to risky sex among emerging adults: The role of trauma-related intrusions and alcohol problems. J. Adolesc. Health.

[73] Seto M.C., Lalumière M.L., Harris G.T., Chivers M.L. (2012). The sexual responses of sexual sadists. J. Abnorm. Psychol..

[74] Sandler J.C., Freeman N.J. (2007). Typology of female sex offenders: A test of Vandiver & Kercher. Sex. Abus..

[75] Denov M.S. (2003). The myth of innocence: Sexual scripts and the recognition of child sexual abuse by female perpetrators. J. Sex Res..

[76] Janus S.S., Janus C.L. (1993). The Janus Report on Sexual Behavior.

[77] Joyal C.C., Carpentier J. (2017). The prevalence of paraphilic interests and behaviors in the general population: A provincial survey. J. Sex Res..

[78] Richters J., Grulich A.E., Visser R.O., Smith A., Rissel C.E. (2003). Sex in Australia: Autoerotic, esoteric, and other sexual practices engaged in by a representative sample of adults. Aust. N. Z. J. Public Health.

[79] Hales S.T., Gannon T.A. (2022). Understanding sexual aggression in UK male university students: An empirical assessment of prevalence and psychological risk factors. Sex. Abus..

[80] Gold S.R., Clegg C.L. (1990). Sexual fantasies of college students with coercive experiences and coercive attitudes. J. Interpers. Violence.

[81] Mann R.E., Hanson R.K., Thornton D. (2010). Assessing risk for sexual recidivism: Some proposals on the nature of psychologically meaningful risk factors. Sex. Abus..

[82] Brouillette-Alarie S., Proulx J., Hanson R.K. (2018). Three central dimensions of sexual recidivism risk: Understanding the latent constructs of Static-99R and Static-2002R. Sex. Abus..

[83] Carvalho J. (2018). Paraphilic sexual interests and sexual offending: Implications for risk assessment and treatment. J. Sex. Med..

[84] Henshaw M., Ogloff J.R.P., Clough J.A. (2017). Looking beyond the screen: A critical review of the literature on the online child pornography offender. Sex. Abus..

[85] Henshaw M., Ogloff J.R.P., Clough J.A. (2018). Demographic, mental health, and offending characteristics of online child exploitation material offenders: A comparison with contact-only and dual sexual offenders. Behav. Sci. Law.

[86] Long M.L., Alison L.A., McManus M.A. (2013). Child pornography and likelihood of contact abuse: A comparison between contact child sexual offenders and noncontact offenders. Sex. Abus..

[87] Wolak J., Finkelhor D., Mitchell K.J. (2011). Child pornography possessors: Trends in offender and case characteristics. Sex. Abus..

[88] Chan H.C.O., Heide K.M., Beauregard E. (2011). What propels sexual murderers: A proposed integrated theory of social learning and routine activities theories. Int. J. Offender Ther. Comp. Criminol..

[89] Chan H.C.O. (2015). Understanding Sexual Homicide Offenders: An Integrated Approach.

[90] Henry O., Mandeville-Norden R., Hayes E., Egan V. (2010). Do internet-based sexual offenders reduce to normal, inadequate and deviant groups?. J. Sex. Aggress..

[91] Neutze J., Seto M.C., Schaefer G.A., Mundt I.A., Beier K.M. (2011). Predictors of child pornography offenses and child sexual abuse in a community sample of pedophiles and hebephiles. Sex. Abus..

[92] Reijnen L., Bulten E., Nijman H. (2009). Demographic and personality characteristics of Internet child pornography downloaders in comparison to other offenders. J. Child Sex. Abus..

[93] van Wijk A., Nieuwenhuis A., Smeltink A. (2009). Een Verkennend Onderzoek Naar Downloaders van Kinderporno [An Exploratory Investigation on Child Pornography Offenders].

[94] Firestone P., Bradford J.M., Greenberg D.M., Larose M.R. (1998). Homicidal sex offenders: Psychological, phallometric, and diagnostic features. J. Am. Acad. Psychiat. Law.

[95] Oliver C.J., Beech A.R., Fishers D., Beckett R. (2007). A comparison of rapists and sexual murderers on demographic and selected psychometric measures. Int. J. Offender Ther. Comp. Criminol..

[96] Vettor S., Beech A.R., Woodhams J., Proulx J., Beauregard E., Lussier P., Leclerc B. Rapists and sexual murderers: Combined pathways to offending. Pathways to Sexual Aggression.

[97] Andres E.B., Choi E.P.H., Fung A.W.C., Lau K.W.C., Ng N.H.T., Yeung M., Johnston J.M. (2021). Comprehensive sexuality education in Hong Kong: Study protocol for process and outcome evaluation. BMC Public Health.

[98] Abbey A., Zawacki T., Buck P.O., Clinton A.M., McAuslan P. (2004). Sexual assault and alcohol consumption: What do we know about their relationship and what types of research are still needed?. Aggress. Violent Behav..

[99] Kraanen F.L., Emmelkamp P.M.G. (2011). Substance misuse and substance use disorders in sex offenders: A review. Clin. Psychol. Rev..

[100] Kingston D.A., Yates P.M., Firestone P. (2012). The self-regulation model of sexual offender treatment: Relationship to risk and need. Law Hum. Behav..

[101] Martens M.P., Page J.C., Mowry E.S., Damann K.M., Taylor K.K., Cimini M.D. (2006). Differences between actual and perceived student norms: An examination of alcohol use, drug use, and sexual behavior. J. Am. Coll. Health.

[102] Kingston D.A., Bradford J.M. (2013). Hypersexuality and recidivism among sexual offenders. Sex. Addict. Compulsivity.

[103] Marshall L.E., Marshall W.L. (2007). Sexual addiction in incarcerated sexual offenders. Sex. Addict. Compulsivity.

